# Supplementary material for: Nucleoid remodeling during environmental adaptation is regulated by HU-dependent DNA bundling
Source: Nat Commun. 2020 Jun 9;11:2905. doi: 10.1038/s41467-020-16724-5 (PMC7283360; doi:10.1038/s41467-020-16724-5)
Supplement: Supplementary file 1 — Supplementary Information [file 41467_2020_16724_MOESM1_ESM.pdf]

Supplementary information

Nucleoid remodeling during environmental adaptation is regulated by HU dependent DNA bundling

Remesh S.G., Verma S. *et al*

This PDF file contains: -

- Supplementary Figures 1-5
- Supplementary Table 1, 2, 3

Supplementary Figure 1

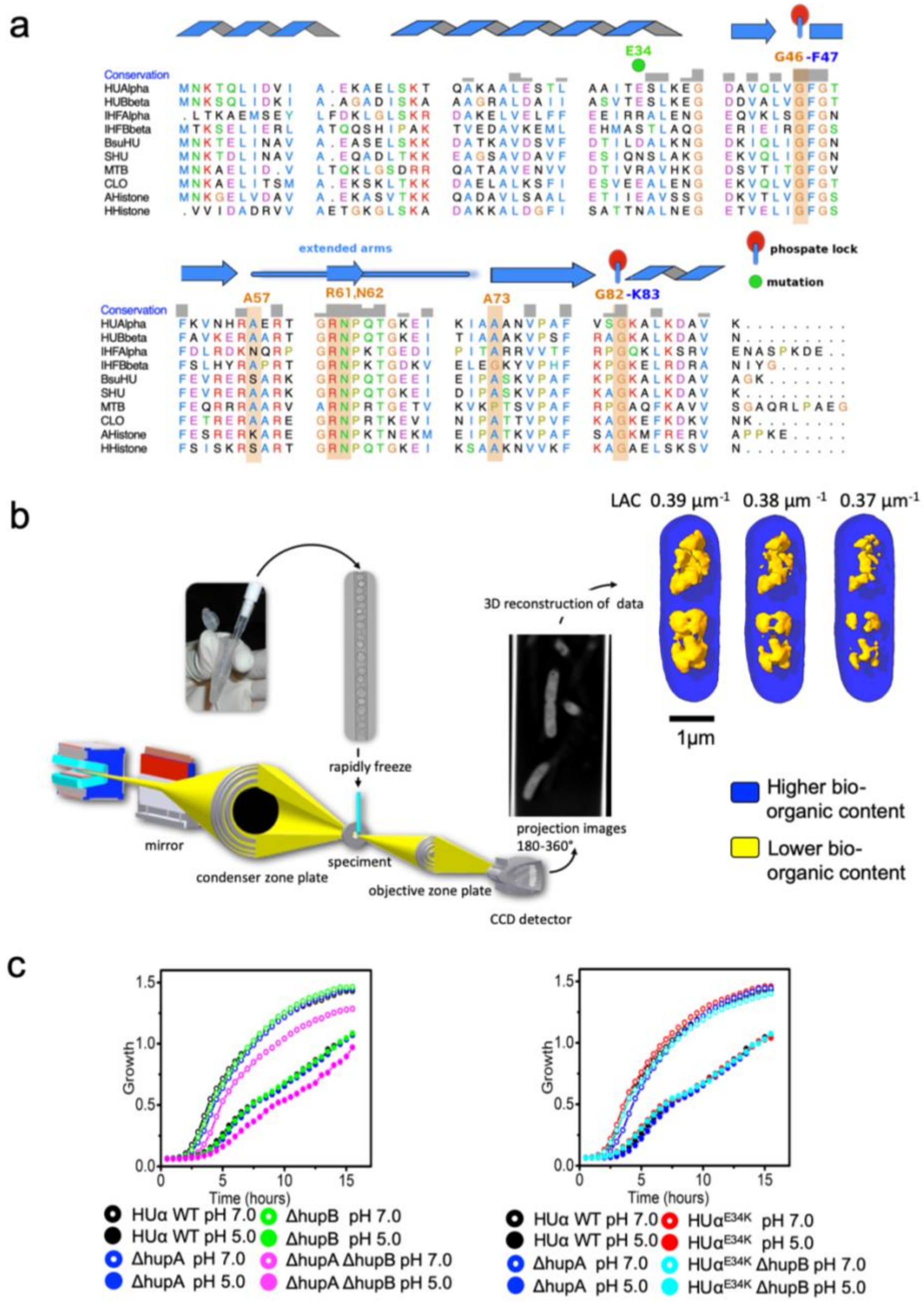

Supplementary Figure 1. Sequence alignment of prokaryotic HU/IHF family members, Soft X-ray tomography based near-native imaging of E coli WT and mutant strains and their growth curves. (a) Sequence alignment of prokaryotic HU/IHF family members is shown here. Residues Gly46, Gly82 and residues in the extended arms – Arg61 and Asn62 (orange box) are strictly conserved across the different HU family members. Residues Gly46 and Gly82 (red dot) of HUαα are critical in forming the phosphate lock with native dsDNA. Residue Lys83 involved in protein-dsDNA interaction is also shown (blue). Position of the point mutation (E34K) are marked with a green dot. Secondary structure elements are shown above the sequence. HUAlpha: *E. coli* HU α-chain; HUBeta: *E. coli* HU β-chain; IHFAlpha: *E. coli* α-chain; IHFBeta: *E. coli* IHF

β-chain; BsuHU: *Bacillus subtilis* HU; SHU: *Staphylococcus aureus* HU; MTB: *Mycobacterium tuberculosis* HU; CLO: *Clostridium saccharolyticum*; AHistone *Anabaena cylindrica* HU; HHistone: *Halorubrum aidingense* HU **(b)** Schematic of the optical layout of the soft X-ray microscope XM-2 at beamline 2.1 at the Advanced Light Source (ALS) at Lawrence Berkeley National lab (adapted from LeGros et al., 2015). A low-field (1.3 T) bend-magnet source at ALS provides the soft X-ray with peak brightness over the important 'water window' wavelength range. The flat mirror acts to cut-off high-energy photons. Fresnal zone plate condenser lens in combination with a central beam stop and a pinhole acts as a linear zone plate monochromator. The specimen is loaded into pre-cut glass capillaries, rapidly frozen using an in-house rapid freezing equipment and transferred to a cryogenic specimen rotation stage for tomographic data collection. Another Fresnal zone plate objective lens transmits a magnified image of the illuminated specimen onto the CCD (charge-coupled device) detector. The projection images collected around a rotation axis in 2° increments are normalized, aligned and tomographic reconstructions calculated using iterative reconstruction methods. Representative 3D reconstructions of an *E. coli* cell segmented at different LAC values are shown. The macrodomain architecture is maintained across the different LAC values though the exact boundaries of individual macrodomains are indeterminate **(c)** Growth curve for *E. coli* WT strain (with functional *hupA* and *hupB*, SCV96) and mutant strains SCV18 (with no functional *hupA)*, SCV19 (with no functional *hupB)*, SCV27 (with no functional *hupA* or *hupB*), SCV56 (hupAE34K with functional *hupB*) and SCV85 (*hupAE34K* with no functional *hupB*) at either pH 7.0 or pH 5.0.

Supplementary Figure 2

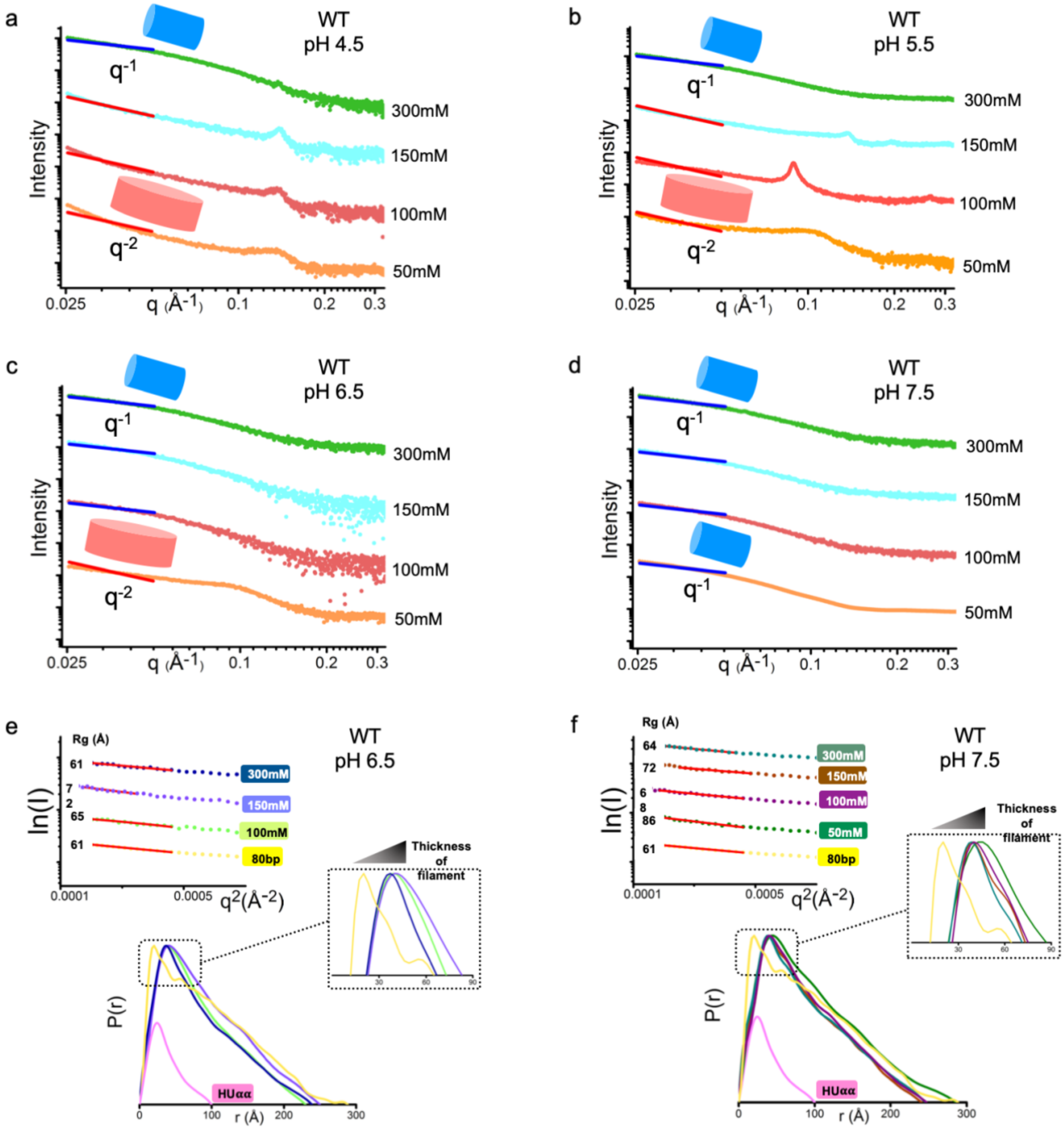

Supplementary Figure 2. Double logarithmic representation of experimental SAXS profiles. (**a-d**) Double logarithmic representation of experimental SAXS profiles for HUαα in complex with 80bp DNA measured at different pH and increasing salt concentrations are shown **(a)** At pH 4.5, the low q region of curves between salt concentrations 50-150 mM NaCl match to the reference line (red) for lamellar structures ($I \propto q^{-2}$) while the curve at high salt concentration (300 mM NaCl) matches the reference line (blue) for tubular/filament-like structures ($I \propto q^{-1}$) **(b)** At pH 5.5**,** the low q region of curves between salt concentrations 50 -100 mM NaCl match to the reference line (red) for lamellar structures ($I \propto q^{-2}$) while the curves at higher salt concentration

(150 – 300 mM NaCl) match the reference line (blue) for tubular/filament-like structures ($I \propto q^{-1}$) (**c**) At pH 6.5, the low q region of curve for salt concentrations 50 mM NaCl match to the reference line (red) for lamellar structures ($I \propto q^{-2}$) while curves at higher salt concentration (100 – 300 mM NaCl) match the reference line (blue) for tubular/filament-like structures ($I \propto q^{-1}$) **(d)** At pH 7.5, the low q region of curve for all the curves match the reference line (blue) for tubular/filament-like structures ($I \propto q^{-1}$) (**e-f**) Guinier regions for the experimental SAXS curves for HUαα in complex with 80bp DNA measured at pH 6.5 and pH 7.5 and increasing salt concentration are also shown and are color coded as indicated. (**upper panels**) Linearity in the Guinier region with the limits $qxR_g<1.3$ (red line) indicates persistence of long filaments and absence of aggregation for protein/DNA complexes at pH 6.5 and pH 7.5. The corresponding pair distribution function normalized to particle volume for protein/DNA complexes at pH 6.5 and pH 7.5 (**lower panels**) indicates that thickness of the filament decreases with increasing salt concentration. P(r) functions also show maximal dimension of the protein/DNA complexes between 250-290Å.

Supplementary Figure 3

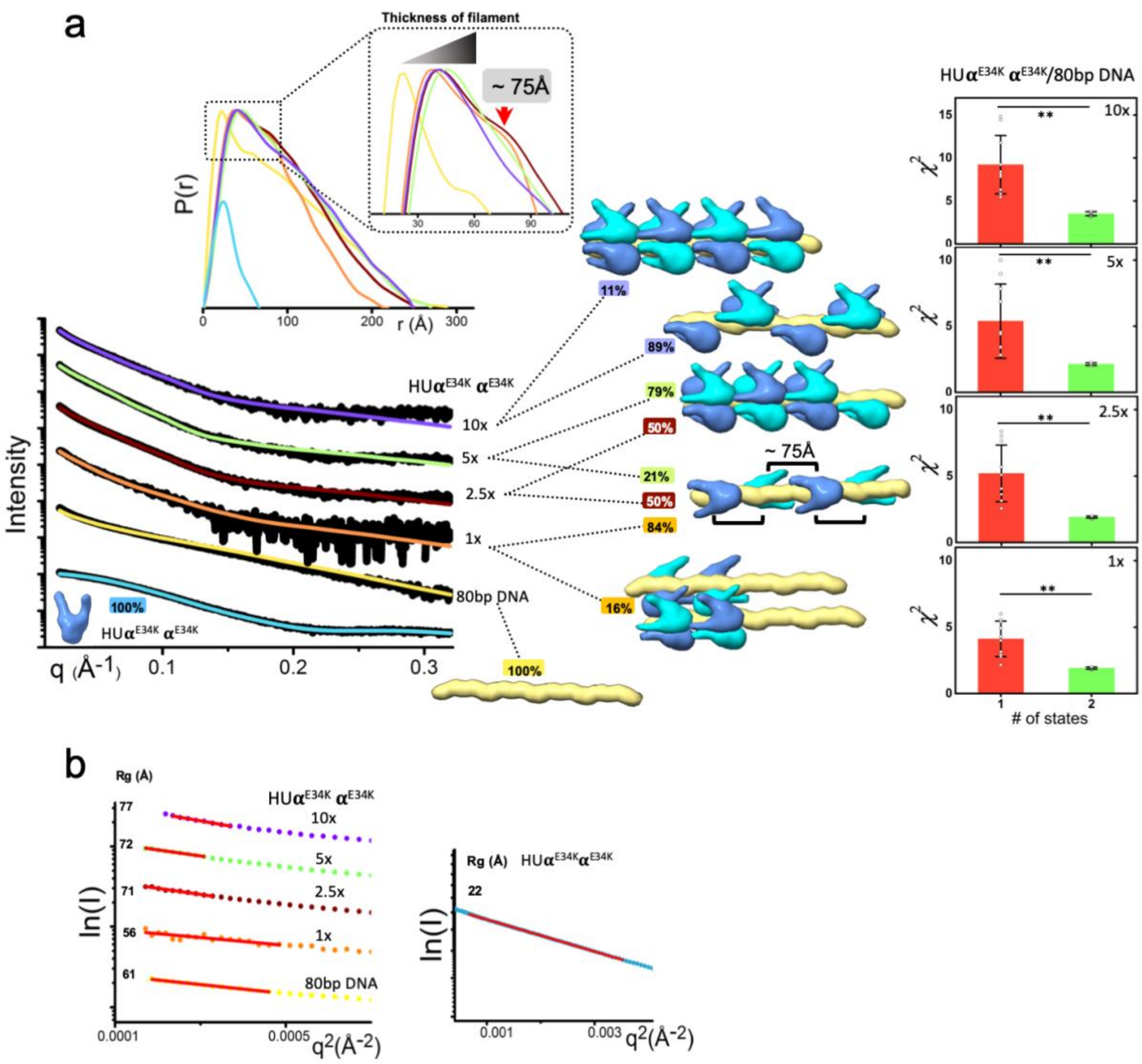

Supplementary Figure 3. HUα mutation and its effects on HUαα-DNA assembly. (a) Experimental SAXS curves for mutant HU$\alpha_{E34K}\alpha_{E34K}$ and HU$\alpha_{E34K}\alpha_{E34K}$ in complex with 80bp DNA measured at DNA:HU$\alpha_{E34K}\alpha_{E34K}$ molar ratio of
1:1 (SASDFR6) https://www.sasbdb.org/data/SASDFR6/ ,
1:2.5 (SASDFS6) https://www.sasbdb.org/data/SASDFS6/ ,
1:5 (SASDFT6) https://www.sasbdb.org/data/SASDFT6/ and
1:10 (SASDFU6) https://www.sasbdb.org/data/SASDFU6/ match theoretical SAXS profiles of atomistic models (χ^2 - 1.8, 1.7, 1.9 and 3.0, respectively). Right panel shows the difference in χ^2 comparing the top 10 single-state and multi-state ensemble (χ^2 data are presented as mean values +/- SD determined from n = 10 independent models for each protein:DNA ratio. The data points are shown as black open circles. Statistical significance was assessed using a two-sided two-sample t-test. All differences between means with $p < 0.01$ are indicated by **). The models were created using multiple asymmetric units of the crystal structure of HU$\alpha_{E34K}\alpha_{E34K}$ - DNA to generate

an $HU\alpha_{E34K}\alpha_{E34K}$ - 80bp DNA nucleoprotein assembly. The models were fit using FoXS/Multi-FoXS. The models are displayed as low-resolution molecular surfaces. Pair distribution (P(r)) functions were calculated from the corresponding SAXS curves and normalized based on experimentally determined volumes of assemblies. The peak around 75Å (inset) corresponds to the protein-protein distance as indicated for DNA:$HU\alpha_{E34K}\alpha_{E34K}$ molar ratio of 1:2.5 With increasing protein concentration the DNA is covered with more protein molecules. **(b)** Guinier regions for the experimental SAXS curves for $HU\alpha_{E34K}\alpha_{E34K}$ and $HU\alpha_{E34K}\alpha_{E34K}$ in complex with 80bp DNA at DNA:$HU\alpha_{E34K}\alpha_{E34K}$ molar ratio of 1:1, 1:2.5, 1:5 and 1:10 are shown and are color coded as indicated. Linearity in the Guinier region with the limits $q*R_g<1.3$ (red line) indicates persistence of long filaments and absence of aggregation in the sample.

Supplementary Figure 4

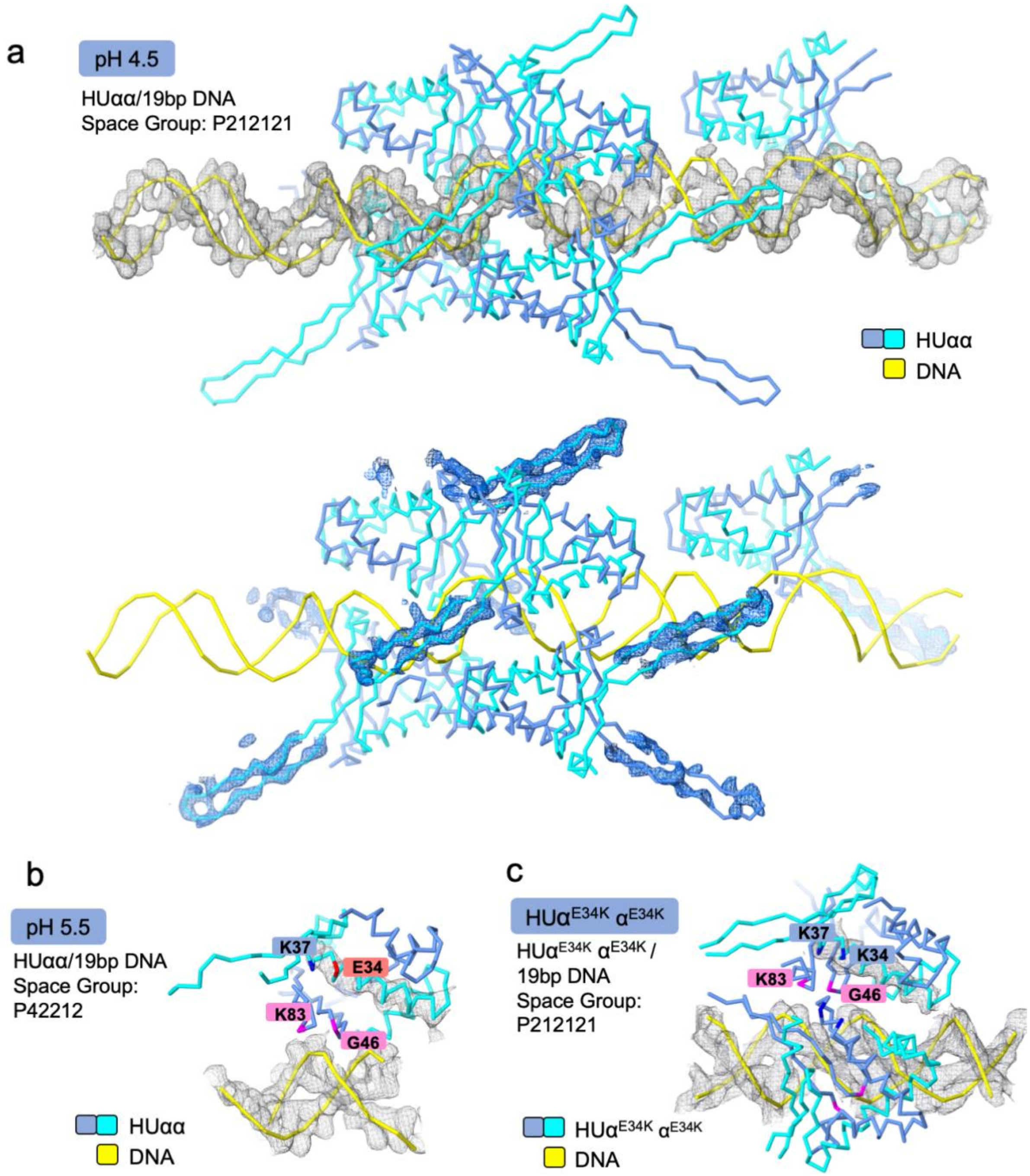

Supplementary Figure 4. Asymmetric unit (ASU) of three protein/DNA complexes. Asymmetric unit (ASU) of three protein/DNA complexes showing 2Fo-Fc electron density map contoured at σ=1.0 for selected regions. **(a)** HUαα-DNA structure at pH 4.5 - **upper panel** – The continuous density for DNA (gray mesh) is shown **lower panel** – Clear density map for arms region of HUαα (residues His54 – Asn75) for 3 molecules in the ASU is shown (**b**) HUαα-DNA at pH 5.5 - Density map for the DNA and selected region Lys18 - Glu38 (gray mesh) is shown **c**. HUαE34KαE34K - DNA density map for the DNA and selected region Lys18 - Glu38 (gray mesh) is shown

Supplementary Figure 5

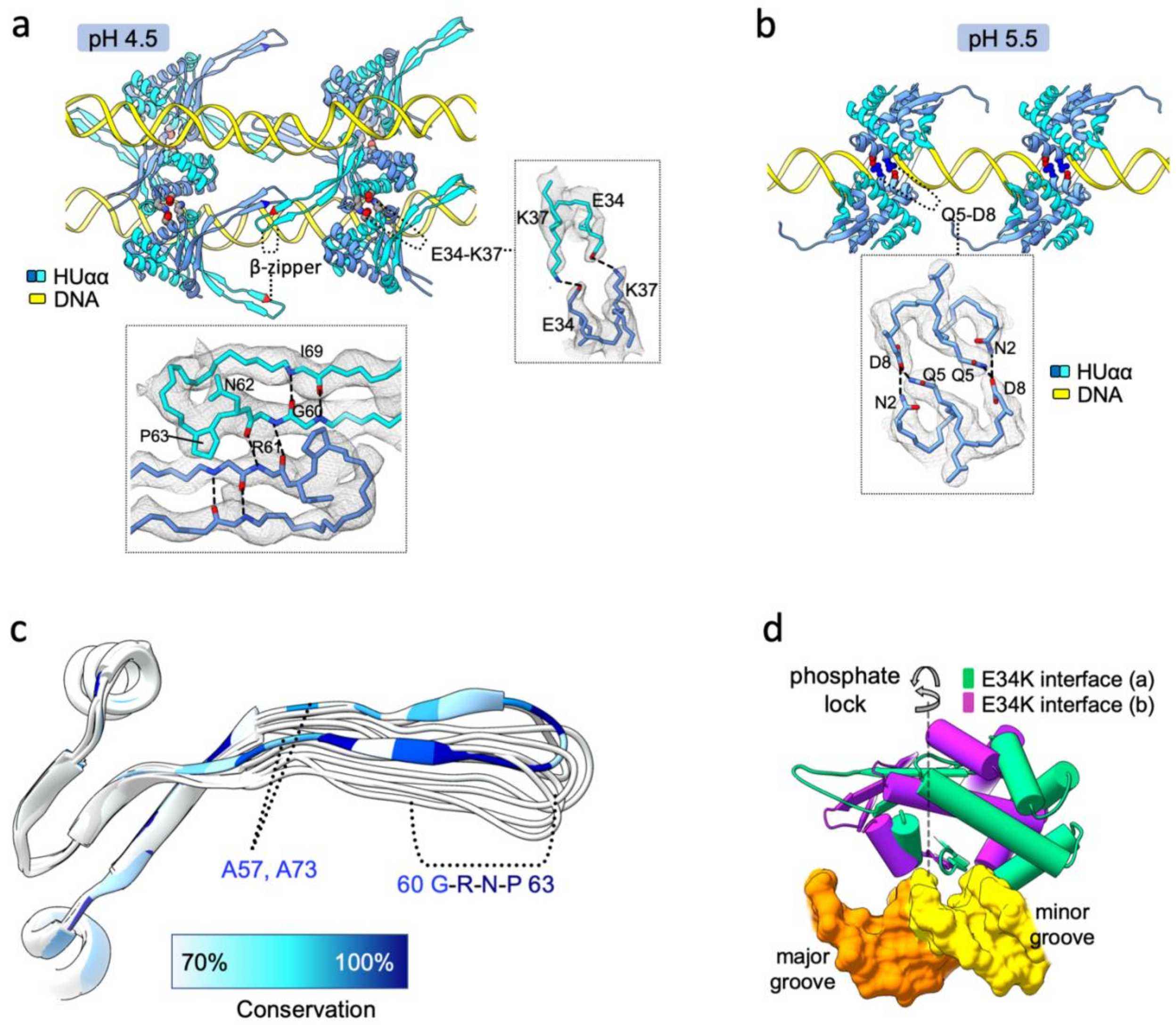

Supplementary Figure 5. HUαα-DNA complexes and role of HUαα residues. (a) Combining different ASU of HUαα-DNA structure at pH 4.5 shows two regions critical for protein-protein interaction. Well-defined electron density of E34 and K37 residues is shown that highlights the intermolecular hydrogen bonds coupling two HUαα dimers. In addition, at low pH, arms of the HUαα dimer bridge oppositely facing dimers located on the adjacent DNA strands through intermolecular backbone hydrogen bonds between highly conserved residues R61 and N62 **(b)** HUαα-DNA structure at pH 5.5 shows no arms coupling or interaction via E34 and K37 hydrogen bonds. Intermolecular hydrogen bonds through residues Gln5 and Asp8 create an alternative coupling of HUαα dimers **(c)** The flexibility of the HUα arms region results in several non-superimposable HUα arms. Six HUαs from one unit cell of the HUαα-DNA, pH 4.5 crystal structure are superimposed and colored by the level of sequence conservations described in the Supplementary Figure 1a. **(d)** The two interfaces between $HU\alpha_{E34K}\alpha_{E34K}$ and DNA in the crystal structure of $HU\alpha_{E34K}\alpha_{E34K}$- DNA are overlaid on the DNA to shows the ball-socket joint around the DNA minor groove similar to wild type HUαα (see Figure 5c).

Supplementary Table 1. X-ray Diffraction data collection and refinement statistics (for Figure 5 and Supplementary Figure 4, 5)

(PDB ID)	**HUαα - DNA pH 4.5** (6O8Q)	**HUαα - DNA pH 5.5** (6O6K)	**HU$\alpha_{E34K}\alpha_{E34K}$ - DNA** (6OAJ)
**Data collection**			
Space Group	P212121	P42212	P212121
Cell Dimensions			
a, b, c (Å)	59.41, 61.15, 351.20	84.79, 84.79, 63.33	64.60. 86.35, 91.69
α, β, γ (°)	90, 90, 90	90, 90, 90	90, 90, 90
Resolution range (Å)	50.18- 3.21 (3.33-3.21)	59.96-3.60 (3.73-3.60)	40.49- 4.09 (4.24- 4.09)
R_{meas}	0.1852 (1.444)	0.08385 (0.6364)	0.1316 (2.129)
R_{pim}	0.0531 (0.3944)	0.01722 (0.1253)	0.05499 (0.8626)
I/σ	11.31(1.61)	33.10 (6.57)	7.28 (1.01)
Redundancy	12.3 (13.1)	24.2 (25.5)	6.2 (6.0)
Number of unique reflections	20,484 (1608)	2939 (286)	4331 (407)
Completeness (%)	93.91 (73.05)	99.90 (100.00)	99.29 (98.54)
Wilson B-factor	88.11	118.71	209.21
$CC_{1/2}$	0.999 (0.83)	1.000 (0.959)	0.996 (0.479)
**Refinement statistics**			
Number of reflections (F>0)	20461 (1545)	2937 (286)	4318 (406)
Maximum resolution (Å)	3.21	3.60	4.09
R_{work}	0.3066 (0.3883)	0.2609 (0.3320)	0.3260 (0.4029)
R_{free}	0.3303 (0.4106)	0.3133 (0.4568)	0.3312 (0.5085)
**Number of atoms**	8361	1474	2993
Protein	7748	1328	2696
DNA	847	146	297
Solvent molecules (waters)	-	-	-
**Ramachandran statistics (%)**			
Favored	95.73	97.83	94.66
Allowed	4.15	2.17	5.34
Outliers	0.12	0.0	0.0
**R.m.s.d. from ideal geometry**			
Bond length (Å)	0.006	0.005	0.005
Bond angles (°)	0.93	0.86	1.02
**Average B values (Å$_2$)**			
Protein	83.74	133.24	90.88
Water molecules	-	-	-
**Number of TLS groups**	-	9	-

A single crystal was used for refinement
*Number in parenthesis refer to highest resolution shell

	HUαα	80bp DNA	HUαα/ 80bp pH 4.5 50mM	HUαα/ 80bp pH 4.5 100mM	HUαα/ 80bp pH 4.5 150mM	HUαα/ 80bp pH 4.5 300mM	HUαα/ 80bp pH 5.5 50mM	HUαα/ 80bp pH 5.5 100mM	HUαα/ 80bp pH 5.5 150mM	HUαα/ 80bp pH 5.5 300mM	HUαα/ 80bp pH 6.5 50mM	HUαα/ 80bp pH 6.5 100mM	HUαα/ 80bp pH 6.5 150mM	HUαα/ 80bp pH 6.5 300mM	HUαα/ 80bp pH 7.5 50mM	HUαα/ 80bp pH 7.5 100mM	HUαα/ 80bp pH 7.5 150mM	HUαα/ 80bp pH 7.5 300mM	HUα$_{E34K}$ α$_{E34K}$	HUα$_{E34K}$ α$_{E34K}$/ 80bp DNA pH 4.5	HUα$_{E34K}$ α$_{E34K}$/ 80bp DNA pH 5.5	HUα$_{E34K}$ α$_{E34K}$/ 80bp DNA pH 6.5	HUα$_{E34K}$ α$_{E34K}$/ 80bp DNA pH 7.5
**SASBDB ID**	SASD FN6 https://www.sasbdb.org/data/SASDFN6/	SASD FP6 https://www.sasbdb.org/data/SASDFP6/	SASD F36 https://www.sasbdb.org/data/SASDF36/	SASD F46 https://www.sasbdb.org/data/SASDF46/	SASD F56 https://www.sasbdb.org/data/SASDF56/	SASD F66 https://www.sasbdb.org/data/SASDF66/	SASD FX5 https://www.sasbdb.org/data/SASDFX5/	SASD FY5 https://www.sasbdb.org/data/SASDFY5/	SASD FZ5 https://www.sasbdb.org/data/SASDFZ5/	SASD F26 https://www.sasbdb.org/data/SASDF26/	SASD FT5 https://www.sasbdb.org/data/SASDFT5/	SASD FU5 https://www.sasbdb.org/data/SASDFU5/	SASD FV5 https://www.sasbdb.org/data/SASDFV5/	SASD FW5 https://www.sasbdb.org/data/SASDFW5/	SASD FP5 https://www.sasbdb.org/data/SASDFP5/	SASD FQ5 https://www.sasbdb.org/data/SASDFQ5/	SASD FR5 https://www.sasbdb.org/data/SASDFR5/	SASD FS5 https://www.sasbdb.org/data/SASDFS5/	SASD FQ6 https://www.sasbdb.org/data/SASDFQ6/	SASD GB3 https://www.sasbdb.org/data/SASDGB3/	SASD GC3 https://www.sasbdb.org/data/SASDGC3/	SASD GD3 https://www.sasbdb.org/data/SASDGD3/	SASD GE3 https://www.sasbdb.org/data/SASDGE3/
**Data Collection**																							
Beamline	SIBYLS beamline 12.3.1																						
Beam energy	11kEV																						
Wavelength (Å)	1.03																						
Sample-detector distance (m)	1.5																						
Detector	Pilatus 2M																						
Exposure time (s)	3s	3s	3s	3s	3s	3s	3s	3s	3s	3s	3s	3s	3s	3s	3s	3s	3s	3s	10s/ 0.1s	10s/0.1s	10s/0.1s	10s/0.1s	10s/0.1s
HU concentration (mg/ml)																							
q range (Å^{-1})	0.01-0.32																						
Temperature (K)	283																						
Structural Parameters																							
I(0) (real) (cm^{-1})	9.3	785	N/A	N/A	N/A	263	N/A	N/A	N/A	158	N/A	91	72	89	94	94	103	106	442	N/A	N/A	262	383
R_g (real) (Å)	29	69	N/A	N/A	N/A	87	N/A	N/A	N/A	69	N/A	70	74	70	92	69	66	71	22	N/A	N/A	78	75
I(0) (reciprocal) (cm^{-1})	7.5	733	N/A	N/A	N/A	264	N/A	N/A	N/A	154	N/A	86	72	83	102	93	92	99	450	N/A	N/A	262	391
R_g (reciprocal) (Å)	30	77	N/A	N/A	N/A	59	N/A	N/A	N/A	64	N/A	62	69	60	89	65	59	63	22	N/A	N/A	73	71
Cross sectional radius R_c (Å)	N/A	10.7	N/A	N/A	N/A	19.5	N/A	N/A	N/A	20.9	N/A	21.1	21.9	18.5	22	21.5	20.7	19	N/A	N/A	N/A	21.4	20.8
D_{max} (Å)$_a$	106	290	N/A	N/A	N/A	274	N/A	N/A	N/A	250	N/A	244	250	285	287	247	241	240	67	N/A	N/A	280	275
Porod Volume (Å^3)	68	238	N/A	N/A	N/A	383	N/A	N/A	N/A	268	N/A	274	352	218	410	336	308	242	36	N/A	N/A	330	309
Calculated monomeric M_r from sequence (kDa)	19	56	19/56	19/56	19/56	19/56	19/56	19/56	19/56	19/56	19/56	19/56	19/56	19/56	19/56	19/56	19/56	19/56	19	19/56	19/56	19/56	19/56
Molecular weight $_b$	37	59	N/A	N/A	N/A	300	N/A	N/A	N/A	230	N/A	220	300	190	360	260	260	190	20	65	69	157	157
**Software**																							
Primary data reduction	SCÅTTER																						
Data processing	Gnom / Scåtter	Gnom / Scåtter	SAS View	SAS View	SAS View	Gnom/ Scåtter	SAS View	SAS View	SAS View	Gnom/ Scåtter	SAS View	Gnom/ Scåtter	Gnom/ Scåtter	Gnom/ Scåtter	Gnom/ Scåtter	Gnom/ Scåtter	Gnom/ Scåtter	Gnom/ Scåtter	Gnom/ Scåtter	Gnom/ Scåtter	Gnom/ Scåtter	Gnom/ Scåtter	Gnom/ Scåtter
Rigid body modelling	FoXS/ Multi FoXS	FoXS	N/A	N/A	N/A	N/A	N/A	N/A	N/A	FoXS	N/A	FoXS	FoXS	FoXS	FoXS	FoXS	FoXS	FoXS	FoXS	N/A	N/A	FoXS	FoXS
χ^2	10.5	129								14.7		4.9	3.2	4.4	3.5	4.6	2.4	7.6	4.0			3.8	4.3
3D graphics representations	CHIMERA																						

$_a$ D_{max} is defined by P(r) function
$_b$ MW estimation calculated using Volume of Correlation

Supplementary Table 2. SAXS data collection and analysis parameters (Related Figure 4 and Supplementary Figures 2 and 3)

Supplementary Table 3. Lamellar stack Caille function model parameters (Related Figure 4 and Supplementary Figure 2)

	Bilayer thickness	Number of layers	d-spacing	Caille parameter	Polydispersity/PD [ratio]	
					Distribution of thickness	Distribution of d-spacing
**HUαα 80bp DNA pH 4.5, 50mM NaCl**	34.88	14.0	42.36	0.27	0.141	0.136
**HUαα 80bp DNA pH 4.5, 100mM NaCl**	35.86	30	41.34	0.34	0.142	0.123
**HUαα 80bp DNA pH 4.5, 150mM NaCl**	40	75	43.76	0.25	0.098	0.04
**HUαα 80bp DNA pH 5.5, 50mM NaCl**	31.32	15	58.00	0.52	0.00	0.15
**HUαα 80bp DNA pH 5.5, 100mM NaCl**	35	25	70.00	0.15	0.00	0.03
**HUαα 80bp DNA pH 5.5, 150mM NaCl**	36.32	24	44.25	0.38	0.007	0.052
**HUαα 80bp DNA pH 6.5, 50mM NaCl**	35.35	8	62	0.55	0.15	0.15
