# Supplementary material for: Nucleoid remodeling during environmental adaptation is regulated by HU-dependent DNA bundling
Source: Nat Commun. 2020 Jun 9;11:2905. doi: 10.1038/s41467-020-16724-5 (PMC7283360; doi:10.1038/s41467-020-16724-5)
Supplement: Supplementary file 3 — Description of Additional Supplementary Files [file 41467_2020_16724_MOESM3_ESM.pdf]

Description of Additional Supplementary Files

File Name: Supplementary Data 1
Description: Differentially expressed genes between lag phase and log phase in WT strain at pH 7.0. Differentially expressed genes were identified using glmTreat (t-test relative to a threshold) function of the *edgeR* package in R using log2 fold change (logFC) value significantly above 1.5 at an false discover rate (FDR) cut-off of 5%. (Related Figure 6)

File Name: Supplementary Data 2
Description: Differentially expressed genes between pH 7.0 and pH 5.0 in WT strain (SCV96). Differentially expressed genes were identified using glmTreat (t-test relative to a threshold) function of the *edgeR* package in R using log2 fold change (logFC) value significantly above 1.5 at an false discover rate (FDR) cut-off of 5%. (Related Figure 6).

File Name: Supplementary Data 3
Description: Differentially expressed genes between pH 7.0 and pH 5.0 in *ΔhupAΔhupB* (SCV27). Differentially expressed genes were identified using glmTreat (t-test relative to a threshold) function of the *edgeR* package in R using log2 fold change (logFC) value significantly above 1.5 at an false discover rate (FDR) cut-off of 5%. (Related Figure 6).

File Name: Supplementary Data 4
Description: Differentially expressed genes between pH 7.0 and pH 5.0 in *hupAE34K* (SCV56). Differentially expressed genes were identified using glmTreat (t-test relative to a threshold) function of the *edgeR* package in R using log2 fold change (logFC) value significantly above 1.5 at an false discover rate (FDR) cut-off of 5%. (Related Figure 6).

File Name: Supplementary Data 5
Description: Differentially expressed genes between pH 7.0 and pH 5.0 in *hupAE34KΔhupB* (SCV85). Differentially expressed genes were identified using glmTreat (ttest relative to a threshold) function of the *edgeR* package in R using log2 fold change (logFC) value significantly above 1.5 at an false discover rate (FDR) cut-off of 5%. (Related Figure 6).

File Name: Supplementary Movie 1
Description:Soft X-ray tomography volumetric 3-dimensionsal (3D) reconstructions show E. *coli* nucleoid region at Lag, exponential and stationary growth phase. Low bio-organic content was segmented at specific LAC value (see Methods) to highlight architecture of nucleoid macrodomains.

File Name: Supplementary Movie 2
Description: A morph between the HUαα-HUαα interface from the crystal structure of HUαα-DNA at the pH 4.5 with interface seen in the crystal structure at the pH 5.5 (top panel) or crystal structure of HU$\alpha^{E34K}\alpha^{E34K}$-DNA (bottom panel) shows decoupling of dimers at pH 5.5 and E34K mutation
