# Supplementary material for: Nucleoid remodeling during environmental adaptation is regulated by HU-dependent DNA bundling
Source: Nat Commun. 2020 Jun 9;11:2905. doi: 10.1038/s41467-020-16724-5 (PMC7283360; doi:10.1038/s41467-020-16724-5)
Supplement: Supplementary file 11 — Reporting Summary [file 41467_2020_16724_MOESM11_ESM.pdf]

nature research

Corresponding author(s): Michal Hammel

Last updated by author(s): Apr 25, 2020

Reporting Summary

Nature Research wishes to improve the reproducibility of the work that we publish. This form provides structure for consistency and transparency in reporting. For further information on Nature Research policies, see Authors & Referees and the Editorial Policy Checklist.

Statistics

For all statistical analyses, confirm that the following items are present in the figure legend, table legend, main text, or Methods section.

n/a	Confirmed	
☐	☒	The exact sample size (*n*) for each experimental group/condition, given as a discrete number and unit of measurement
☐	☒	A statement on whether measurements were taken from distinct samples or whether the same sample was measured repeatedly
☐	☒	The statistical test(s) used AND whether they are one- or two-sided<br>*Only common tests should be described solely by name; describe more complex techniques in the Methods section.*
☒	☐	A description of all covariates tested
☒	☐	A description of any assumptions or corrections, such as tests of normality and adjustment for multiple comparisons
☐	☒	A full description of the statistical parameters including central tendency (e.g. means) or other basic estimates (e.g. regression coefficient) AND variation (e.g. standard deviation) or associated estimates of uncertainty (e.g. confidence intervals)
☐	☒	For null hypothesis testing, the test statistic (e.g. *F*, *t*, *r*) with confidence intervals, effect sizes, degrees of freedom and *P* value noted<br>*Give P values as exact values whenever suitable.*
☒	☐	For Bayesian analysis, information on the choice of priors and Markov chain Monte Carlo settings
☒	☐	For hierarchical and complex designs, identification of the appropriate level for tests and full reporting of outcomes
☒	☐	Estimates of effect sizes (e.g. Cohen's *d*, Pearson's *r*), indicating how they were calculated

Our web collection on statistics for biologists contains articles on many of the points above.

Software and code

Policy information about availability of computer code

Data collection	Beamline 12.3.1 (ALS) - Blu-Ice, Classen et al., 2013 (https://www.ncbi.nlm.nih.gov/pubmed/23396808); Beamline 2.1 (ALS) - XM-2 beamline control GUI on LabView 2012; Beamline 8.3.1 (ALS) - Beamline control GUI Blue-Ice 5; HighSeq Control software (HCS) HD 3.4.0
Data analysis	AREC-3D package (Parkinson et al., 2012); AMIRA® 6 FEI Visualization Sciences Group (http://www.amira.com); Cutadapt (Martin, M., 2011) (https://cutadapt.readthedocs.io/en/stable/); STAR (Dobin, A. et al., 2013) (https://github.com/alexdobin/STAR/releases/tag/2.7.3a); RSEM (Li, B et al., 2011) (https://github.com/deweylab/RSEM); XDS VERSION Jan 26, 2018 (Kabsch, W., 2010) (http://xds.mpimf-heidelberg.mpg.de/); PHASER (McCoy et al., 2010) (http://www.phenix-online.org/); Phenix Refine (Afonine et al., 2012) (http://www.phenix-online.org/); Phenix version 1.13-2998; Coot 0.8.9.1 EL (Emsley et al., 2004) (https://www2.mrc-lmb.cam.ac.uk/personal/pemsley/coot/); PDB2PQR version 2.1.1 (Dolinsky et al., 2004) (http://nbcr-222.ucsd.edu/pdb2pqr_2.1.1/); SCÅTTER Developed by Dr. Robert Rambo at SIBYLS, now at the Diamond Light Source (Didcot, UK) (http://www.bioisis.net/tutorial/9); GNOM (Svergun, D. et al., 1992) (https://www.embl-hamburg.de/biosaxs/gnom.html); MODELLER (Sali and Blundell, 1993) (https://salilab.org/modeller/) launched from CHIMERA; SasView 4.2.0 (http://www.sasview.org/); FoXS (Schneidman-Duhovny et al., 2013) (https://modbase.compbio.ucsf.edu/foxs/); Multi-FoXS (Schneidman-Duhovny et al., 2016) (https://modbase.comapbio.ucsf.edu/multifoxs/); CHIMERA (Pettersen et al., 2004) (https://www.cgl.ucsf.edu/chimera/); OriginLab 2016 Origin (OriginLab, Northampton, MA) (https://www.originlab.com/); glmTREAT (https://www.rdocumentation.org/packages/edgeR/versions/3.14.0/topics/glmTreat)

For manuscripts utilizing custom algorithms or software that are central to the research but not yet described in published literature, software must be made available to editors/reviewers. We strongly encourage code deposition in a community repository (e.g. GitHub). See the Nature Research guidelines for submitting code & software for further information.

Data

Policy information about availability of data

All manuscripts must include a data availability statement. This statement should provide the following information, where applicable:

- Accession codes, unique identifiers, or web links for publicly available datasets
- A list of figures that have associated raw data
- A description of any restrictions on data availability

Atomic coordinates and structure factors for the crystal structures are deposited in the Protein Data Bank (PDB - (https://www.rcsb.org/)) PDBID: 6O8Q [https://www.rcsb.org/structure/6O8Q], 6O6K [https://www.rcsb.org/structure/6O6K] and 6OAJ [https://www.rcsb.org/structure/6OAJ] (Figure 5 and Supplementary Figure 4,5). SAXS data are deposited in the Small Angle Scattering Biological Data Bank (SASBDB - (https://www.sasbdb.org/)) and SASBDD IDs are listed in the Supplementary Table 2 and figure legend of Supplementary Figure 3 (Figure 4 and Supplementary Figure 2,3) . Soft X-ray tomography data are available here [https://ncxt-nas1.lbl.gov:5001/fsdownload/XKnLbGVgY/Nature%20comm] and will also be made available by the corresponding author on request (Figure 1,2,3). The RNA-Seq are deposited in the Gene Expression Omnibus database (GEO – (https://www.ncbi.nlm.nih.gov/geo/)) with accession code GSE134667 [https://www.ncbi.nlm.nih.gov/geo/query/acc.cgi?acc=GSE134667] (Figure 6).

Field-specific reporting

Please select the one below that is the best fit for your research. If you are not sure, read the appropriate sections before making your selection.

☒ Life sciences ☐ Behavioural & social sciences ☐ Ecological, evolutionary & environmental sciences

For a reference copy of the document with all sections, see nature.com/documents/nr-reporting-summary-flat.pdf

Life sciences study design

All studies must disclose on these points even when the disclosure is negative.

Sample size	Not determined. Manuscript pertains to structural studies and determination of sample size is not required.
Data exclusions	No data excluded
Replication	Cell growth assay - data derived from n = 3 biological replicates. Transcription profiling - data derived from n = 3 biological replicates. Statistics for LAC-Histogram determined from n=10 independent bacterial cell for each experimental condition. SAXS experiments repeated 2/3 times independently. We confirm that all attempts to replicate experiments were successful.
Randomization	N/A. Manuscript pertains to structural studies and use of randomization is not required.
Blinding	N/A. Manuscript pertains to structural studies and use of blinding is not required.

Reporting for specific materials, systems and methods

We require information from authors about some types of materials, experimental systems and methods used in many studies. Here, indicate whether each material, system or method listed is relevant to your study. If you are not sure if a list item applies to your research, read the appropriate section before selecting a response.

Materials & experimental systems

n/a	Involved in the study	
☒	☐	Antibodies
☒	☐	Eukaryotic cell lines
☒	☐	Palaeontology
☒	☐	Animals and other organisms
☒	☐	Human research participants
☒	☐	Clinical data

Methods

n/a	Involved in the study	
☒	☐	ChIP-seq
☒	☐	Flow cytometry
☒	☐	MRI-based neuroimaging
